# Variation in quality of primary-care services in Kenya, Malawi, Namibia, Rwanda, Senegal, Uganda and the United Republic of Tanzania

**DOI:** 10.2471/BLT.16.175869

**Published:** 2017-05-09

**Authors:** Margaret E Kruk, Adanna Chukwuma, Godfrey Mbaruku, Hannah H Leslie

**Affiliations:** a Department of Global Health and Population, Harvard T.H. Chan School of Public Health, 665 Huntington Ave., Boston, MA 02115, United States of America.; b Ifakara Health Institute, Dar es Salaam, United Republic of Tanzania.

## Abstract

**Objective:**

To analyse factors affecting variations in the observed quality of antenatal and sick-child care in primary-care facilities in seven African countries.

**Methods:**

We pooled nationally representative data from service provision assessment surveys of health facilities in Kenya, Malawi, Namibia, Rwanda, Senegal, Uganda and the United Republic of Tanzania (survey year range: 2006–2014). Based on World Health Organization protocols, we created indices of process quality for antenatal care (first visits) and for sick-child visits. We assessed national, facility, provider and patient factors that might explain variations in quality of care, using separate multilevel regression models of quality for each service.

**Findings:**

Data were available for 2594 and 11  402 observations of clinical consultations for antenatal care and sick children, respectively. Overall, health-care providers performed a mean of 62.2% (interquartile range, IQR: 50.0 to 75.0) of eight recommended antenatal care actions and 54.5% (IQR: 33.3 to 66.7) of nine sick-child care actions at observed visits. Quality of antenatal care was higher in better-staffed and -equipped facilities and lower for physicians and clinical officers than nurses. Experienced providers and those in better-managed facilities provided higher quality sick-child care, with no differences between physicians and nurses or between better- and less-equipped clinics. Private facilities outperformed public facilities. Country differences were more influential in explaining variance in quality than all other factors combined.

**Conclusion:**

The quality of two essential primary-care services for women and children was weak and varied across and within the countries. Analysis of reasons for variations in quality could identify strategies for improving care.

## Résumé

**Objectif:**

Analyser les facteurs qui affectent les variations de qualité observées concernant les soins prénatals et aux enfants malades dans des établissements de soins primaires de sept pays africains.

**Méthodes:**

Nous avons rassemblé des données représentatives à l'échelle nationale provenant d'enquêtes évaluant la qualité des services fournis par des établissements de santé situés au Kenya, au Malawi, en Namibie, en Ouganda, en République-Unie de Tanzanie, au Rwanda et au Sénégal (années couvertes par les enquêtes: 2006-2014). Nous avons créé des indices de qualité du processus pour les soins prénatals (premières visites) et les visites aux enfants malades en nous appuyant sur les protocoles de l'Organisation mondiale de la Santé. À l'aide de différents modèles de régression multiniveaux de qualité pour chaque service, nous avons évalué des facteurs liés aux pays, aux établissements, aux prestataires et aux patients susceptibles d'expliquer les variations qualitatives des soins.

**Résultats:**

Nous avons disposé de données tirées de 2594 observations de consultations cliniques pour des soins prénatals et de 11 402 observations de consultations cliniques pour des enfants malades. Dans l'ensemble, les prestataires de soins de santé ont réalisé en moyenne 62,2% (intervalle interquartile, IQR: de 50,0 à 75,0) des huit actions de soins prénatals recommandées et 54,5% (IQR: de 33,3 à 66,7) des neuf actions de soins aux enfants malades lors des visites observées. Les soins prénatals étaient de meilleure qualité dans les établissements mieux dotés en personnel et mieux équipés et ceux fournis par les médecins et les cliniciens se sont révélés de moins bonne qualité que ceux des infirmiers. Les prestataires expérimentés ainsi que ceux qui exerçaient dans des établissements mieux gérés ont fourni des soins de meilleure qualité aux enfants malades, aucune différence n'ayant été observée entre les médecins ou les infirmiers ou entre les centres de consultation mieux équipés et ceux moins bien équipés. Les établissements privés ont obtenu de meilleurs résultats que les établissements publics. Les différences entre les pays ont davantage permis d'expliquer les variations de qualité que tous les autres facteurs combinés.

**Conclusion:**

Deux services de soins primaires essentiels pour les femmes et les enfants se sont révélés de qualité médiocre, celle-ci variant selon les pays et au sein des pays. Une analyse des raisons des variations qualitatives permettrait de définir des stratégies afin d'améliorer les soins.

## Resumen

**Objetivo:**

Analizar los factores que afectan a las variaciones de la calidad observada en la atención prenatal y a niños enfermos en centros de atención primaria de siete países africanos.

**Métodos:**

Se recopilaron datos representativos a nivel nacional de encuestas de evaluación sobre prestación de servicios de centros sanitarios de Kenya, Malawi, Namibia, la República Unida de Tanzanía, Rwanda, Senegal y Uganda (rango anual de encuestas: 2006–2014). En base a los protocolos de la Organización Mundial de la Salud, se crearon índices de calidad de procesos para la atención prenatal (primeras consultas) y para las consultas a niños enfermos. Se evaluaron los factores nacionales, de centros, de proveedores y de pacientes que pudieran explicar las variaciones en la calidad de la atención mediante el uso de distintos modelos de regresión en múltiples niveles de calidad para cada servicio.

**Resultados:**

Se disponía de los resultados para 2 594 y 11 402 observaciones de consultas médicas de atención prenatal y atención a niños enfermos, respectivamente. En general, los profesionales sanitarios realizaron una media de 62,2% (rango intercuartílico, ICR: 50,0 a 75,0) de ocho acciones de atención prenatal recomendadas y de 54,5% (ICR: 33,3 a 66,7) de nueve acciones de atención a niños enfermos en las visitas observadas. La calidad de la atención prenatal fue mejor en los centros con mejor personal y mejores equipos e inferior para los médicos y asistentes clínicos que para los enfermeros. Los proveedores con experiencia y aquellos en centros con una mejor gestión ofrecieron una atención a niños enfermos de mejor calidad, sin diferencias entre médicos y enfermeros o entre centros mejor o peor equipados. Los centros privados superaron la calidad de los públicos. Las diferencias entre países tuvieron un papel más influyente a la hora de explicar la varianza en la calidad que el resto de factores juntos.

**Conclusión:**

La calidad de dos servicios de atención primaria básicos para mujeres y niños era escasa y diversa entre países y dentro de ellos. El análisis de las razones de las variaciones en la calidad podría identificar estrategias para mejorar la atención.

## ملخص

الغرض تحليل العوامل المؤثرة على التباين فيما يخضع للملاحظة من جودة الرعاية المقدمة في مرحلة ما قبل الولادة والرعاية المقدمة للمرضى من الأطفال داخل منشآت الرعاية الأولية في سبع دول أفريقية.

الطريقة جمعنا بيانات تمثيلية على المستوى الوطني مستمدة من مسوح تقييمية للمنشآت الصحية من حيث توفير الخدمة في أوغندا وجمهورية تنزانيا المتحدة ورواندا والسنغال وكينيا وملاوي وناميبيا (المدى الزمني لسنوات المسح: 2006-2014). ووفقًا لبروتوكولات منظمة الصحة العالمية، أنشأنا مؤشرات لجودة تدابير الرعاية المقدمة في مرحلة ما قبل الولادة (الزيارات الأولى) والزيارات الخاصة بالمرضى من الأطفال. وقيَّمنا العوامل الوطنية والعوامل المرتبطة بالمنشأة والمرضى والجهات الموفرة للخدمة والتي قد تفسر التباين في مستوى جودة الرعاية المقدمة، حيث استخدمنا نماذج تحوّف منفصلة متعددة المستويات فيما يتعلق بمستوى الجودة لكل نوع من أنواع الخدمات.

النتائج كانت البيانات متاحةً بشأن 2594 و11402 من عمليات ملاحظة الاستشارات العلاجية فيما يتعلق بالرعاية المقدمة في مرحلة ما قبل الولادة والمقدمة للمرضى من الأطفال، على التوالي. وإجمالاً، بلغ متوسط تنفيذ جهات توفير الرعاية الصحية للإجراءات الموصى بها 62.2‏% (المدى الربيعي: من 50.0 إلى 75.0) وذلك من إجمالي ثمانية إجراءات موصى بها لتقديم الرعاية في مرحلة ما قبل الولادة، و54.5‏% (المدى الربيعي: من 33.3 إلى 66.7) من إجمالي تسع إجراءات للرعاية في الزيارات التي خضعت للملاحظة. وارتفع مستوى جودة الرعاية المقدمة في مرحلة ما قبل الولادة لدى المنشآت التي تحظى بتجهيزات وفريق عمل أكثر كفاءة، بينما انخفض مستوى الجودة لدى الأطباء والمساعدين الإكلينيكيين مقارنةً بالممرضات. وتولى مقدمو الخدمات الصحية من ذوي الخبرة والعاملون في المنشآت التي تحظى بمستوى أفضل من الإدارة تقديم مستوى أعلى من جودة الرعاية المقدمة للمرضى من الأطفال، دون أي اختلاف بين الأطباء والممرضات أو بين العيادات التي يرتفع فيها مستوى التجهيزات وتلك التي يقل فيها مستوى التجهيزات. وتفوقت المنشآت الخاصة في أدائها على المنشآت الحكومية. ولعبت الاختلافات بين الدول دورًا أكثر فعالية في تفسير التباين في مستوى الجودة مقارنةً بالعوامل الأخرى مجتمعةً.

الاستنتاج إن جودة اثنين من أنواع الخدمات الأساسية للرعاية الصحية الأولية المقدمة للنساء والأطفال كانت ضعيفة المستوى ومتباينة فيما بين الدول وفيما بين المناطق داخل الدول. ويمكن أن يؤدي تحليل أسباب التباين في مستوى الجودة إلى تحديد استراتيجيات لتحسين الرعاية المقدمة.

## 摘要

**目的:**

旨在分析影响七个非洲国家初级医疗机构中所观测的产前及患儿护理质量的变化的因素。

**方法:**

我们通过肯尼亚、卢旺达、马拉维、纳米比亚、塞内加尔、坦桑尼亚联合共和国和乌干达的卫生设施服务提供评估调查收集了具有全国代表性的数据(调查年份: 2006–2014)。 根据世界卫生组织协议,我们创建了产前护理(初诊)及患儿就诊流程的质量指标。 我们通过各服务质量的独立多级回归模型对可阐明护理质量变化的全国性、设施、提供者及患者因素进行了评估。

**结果:**

数据分别适用于 2594 及 11 402 份产前护理及患儿临床咨询的观测报告。 总体来说,在所观测的就诊期间,八项推荐产前护理行为中,62.2% (四分差,IQR: 50.0 至 75.0)由医疗护理提供者开展;九项患儿护理行为中,54.5% (IQR: 33.3 至 66.7)由医疗护理提供者开展。 人员及设施配备更为齐全出色的机构产前护理质量会更高一些,且医生及临床助理医师提供的产前护理质量低于护士提供的产前护理质量。经验丰富的提供者以及管理良好的机构中的提供者能够提供更高质量的患儿护理,且护理质量在医生及护士之间或设施配备多与少的诊所之间并无差别。 私营机构表现优于公共机构。 国家差异较所有其它因素而言,在阐明质量差异方面更具影响力。

**结论:**

各国妇女及儿童的两项基本初级医疗服务的质量较为薄弱且在国家之间以及国家内部存在差异。 可通过分析质量变化的原因制定改善护理的策略。

## Резюме

**Цель:**

Проанализировать факторы, обуславливающие различия в качестве дородового наблюдения и ухода за больными детьми в учреждениях первичной медико-санитарной помощи в семи африканских странах.

**Методы:**

Авторы объединили репрезентативные на национальном уровне данные обзорных исследований по оценке предоставления услуг в медицинских учреждениях в Кении, Малави, Намибии, Объединенной Республике Танзания, Руанде, Сенегале и Уганде (учитывались обследования, проводимые в 2006–2014 гг.). На основании протоколов Всемирной организации здравоохранения были разработаны показатели качества для дородового наблюдения (первых визитов) и для ухода за больными детьми. Авторы провели оценку национальных факторов, а также факторов, связанных с медицинским центром, поставщиком медицинских услуг и пациентом, которые могли бы объяснить различия в качестве медицинской помощи, используя отдельные многоуровневые регрессионные модели качества для каждой услуги.

**Результаты:**

Были доступны данные для 2594 и 11 402 обзорных исследований клинических консультаций по дородовому уходу и уходу за больными детьми соответственно. Медицинские работники провели в среднем 62,2% (межквартильный размах, МКР: от 50,0 до 75,0) из восьми рекомендуемых мероприятий по дородовому уходу и 54,5% (МКР: от 33,3 до 66,7) из девяти мероприятий по уходу за больными детьми во время посещений, совершенных под наблюдением исследователей. Качество дородового ухода было выше в медицинских учреждениях, которые были лучше укомплектованы персоналом и оборудованием, и ниже у врачей, чем у медсестер. Опытные поставщики медицинских услуг в медицинских учреждениях с лучшей системой управления обеспечивали уход за больными детьми более высокого качества без каких-либо различий между врачами и медсестрами или между клиниками с лучшим и худшим оснащением. Частные медицинские учреждения превзошли общественные медицинские учреждения. Страновые особенности были более значимы при объяснении различий в качестве, чем все остальные факторы, вместе взятые.

**Вывод:**

Качество двух основных услуг первичной медико-санитарной помощи для женщин и детей было низким и варьировалось как между странами, так и внутри отдельной страны. Анализ причин различий в качестве позволил бы определить стратегии для усовершенствования медико-санитарной помощи.

## Introduction

Although substantial progress has been made in reducing child and maternal deaths in the past 15 years, many women and children in low- and middle-income countries continue to die of avertable causes.^1^ To stimulate a concerted effort to narrow the gap between rich and poor countries, the United Nation’s sustainable development goals (SDGs) include new targets to reduce maternal mortality to less than 70 per 100 000 live births and to reduce deaths of children younger than five years to 25 per 1000 live births by 2030.^2^

The global strategy to date has been to promote higher utilization of health services to treat the diseases that contribute most to mortality among children and women.^3^ Integrated management of childhood illness – a simplified approach for diagnosing and treating malaria, diarrhoea and pneumonia – is one such strategy.^4^^,^^5^ Another approach is antenatal care, which can provide important health benefits to the pregnant woman (e.g. malaria treatment and diagnosis of human immunodeficiency virus infection) and her child (e.g. tetanus toxoid vaccination).^6^ Coverage of these and other essential health services is increasing,^1^ aided by global initiatives to measure and compare coverage across countries, such as the Countdown to 2015 initiative: a multi-country collaboration to collect and publish comparable data.^7^ These data have informed programmes to promote utilization of health care, by providing information, insurance schemes and utilization incentives for communities, among other means.^8^^–^^10^

Less is known about the quality of health services received by women and children when they reach a health-care facility. Some studies have pointed to quality deficits in the delivery of basic maternal and child-health services.^11^^–^^14^ However, unlike for coverage, there is no systematic examination of health-care quality that would permit benchmarking and tracking of progress over time. This is increasingly important as there is renewed interest in strengthening the role of integrated primary care in countries where investments have predominantly targeted communicable diseases.^15^^–^^18^ A functioning primary-care service offers continuous care via preventive and curative services^18^ and is therefore well-positioned to deal with the double burden of infectious and chronic diseases now facing low- and middle-income countries. While primary-care performance is regularly measured in wealthier countries, there are almost no data from lower-income regions.^19^^,^^20^

In this paper, we analyse the variation in the quality of processes of care in health facilities in seven countries in sub-Saharan Africa for two primary-care services: (i) antenatal care and (ii) care of sick children, using observations of clinical care, a gold standard measure of process quality. The results will inform policy-makers about current performance and provide a starting point for a broader discussion of quality measurement in the SDG era.

## Methods

### Study sample

The study sample was drawn from service provision assessment surveys conducted by the demographic and health survey programme. The surveys include four instruments: audits of service readiness in health-care facilities; interviews with health-care providers; direct observations of consultations; and exit interviews with patients. We focused on sub-Saharan Africa and included all surveys between 2006 and 2014 that had data on observations of antenatal and sick-child care (Kenya, 2010; Malawi, 2013; Namibia, 2009; Rwanda, 2007; Senegal, 2012–2014; Uganda, 2007; the United Republic of Tanzania, 2006; Table 1). These surveys use nationally representative samples, or censuses or near censuses (in Malawi, Namibia and Rwanda), of the country’s health facilities.^24^^–^^31^ The resulting data provide the most detailed, nationally representative information available on primary-care quality.

**Table 1:** Demographic and health context in Kenya, Malawi, Namibia, Rwanda, Senegal, Uganda and United Republic of Tanzania

Variable	Year^a^	Country						
		Kenya	Malawi	Namibia	Rwanda	Senegal	Uganda	United Republic of Tanzania
Population, no.	2015	46 050 302	17 215 232	2 458 830	11 609 666	15 129 273	39 032 383	53 470 420
GDP per capita, US$	2015	1 246	255	5 693	638	1 067	572	695
Physicians per 100 000 population, no.	2010–2013	20	2	37	6	6	12	3
Health spending per capita, US$	2014	78	24	499	52	50	59	52
Out-of-pocket spending, % of all health-care spending	2014	26	13	7	28	37	41	23
Crude birth rate per 1000 population	2014	35	39	30	32	38	43	39
Maternal mortality rate per 100 000 live births	2010–2015	510	634	265	290	315	343	398
Under-5 mortality rate per 1000 live births	2015	49	64	45	42	47	55	49
Women aged 15–49 years with at least one antenatal care visit, % of recently pregnant women	2008–2014	96	96	97	99	96	93	88
Children aged < 5 years with respiratory infection, % taken to health facility	2010–2014	66	68	72	50	53	79	31

GDP: gross domestic product; US$: United States dollars.

^a^ Ranges indicate different dates for data in different countries.

Sources: most recent data available from World Bank, World Health Organization, and demographic and health surveys.^21^^–^^23^

Within health-care facilities, up to five clients per provider per clinical area were selected for observation using systematic random sampling. Observers, who were specially trained researchers, assessed: (i) first visits or follow-up visits for antenatal care; and (ii) consultations for children aged five years or younger presenting with illness.

For this analysis we selected data from all primary-care facilities, defined as any facility that was not designated as a hospital by the country. The antenatal care analysis was restricted to first visits, as those had substantially more of the recommended clinical content than did follow-up visits. We calculated sampling weights for each observed visit to adjust for different likelihood of facility and patient selection into the sample. The final weighted results are approximately representative of women and children seen in the health system during the survey timeframe.

### Study outcomes

Using guidelines from the World Health Organization (WHO),^32^^,^^33^ we identified essential elements of clinical care for mothers and infants, and matched these to the indicators available in the service provision assessment surveys. We then created composite quality indices of clinical care for the two services. Each index had items covering history-taking, physical examination, diagnosis, and counselling and management actions that should be done for all patients, regardless of the reason for presentation or the local epidemiology. There were eight items for antenatal care and nine for sick-child care (Table 2). We calculated the percentage of items fulfilled per visit, to provide a continuous quality process score scaled from 0 to 100, whereby a higher score corresponded to greater adherence to the recommended clinical actions.

**Table 2:** Components of clinical quality indices for antenatal and sick-child care services

**Type of service**	**Clinical action by health-care provider**
**Antenatal care**	
History	– Asks ≥ 1 question on pregnancy history^a^ – Asks ≥ 1 question about danger signs in pregnancy
Examination	– Measures blood pressure – Measures weight
Diagnostic tests	– Performs or refers for anaemia test – Performs or refers for urine test
Counselling and management	– Prescribes or gives tetanus toxoid injection – Counsels about danger signs in pregnancy
**Sick-child care**	
History	– Asks ≥ 1 question on infant feeding or drinking – Asks about diarrhoea or vomitin – Asks about fever or seizures – Asks about cough
Examination	– Measures weight – Measures temperature
Counselling and management	– States diagnosis – Counsels about food intake – Counsels about danger signs for return consultation

^a^ Excluding primiparous women.

### Covariates

To construct an explanatory model for observed quality, we drew on Rowe’s framework for explaining the performance of health-care workers.^34^ This framework includes factors related to patients, providers and facilities, as well as the broader health system and community context.^34^ We identified covariates in the data that corresponded to the key constructs in the Rowe framework at the visit, provider, facility and country levels.

Visit-level covariates included: patient’s age (teenage woman [age < 20 years] at antenatal care visits; infant [age < 12 months] at sick-child visits), educational attainment of the caregiver present at the visit, and case complexity (late first visit [≥ 24 weeks gestation] for antenatal care; number of complaints for sick children). Patient-level data came from patient exit interviews and observations. We identified afternoon visits to assess the influence of time of day on provider performance.

For health-care providers, three measures were available: cadre (physician, nurse, or nursing assistant/other), experience (completed preservice training > 5 years previously) and supportive environment. Physicians included medical doctors as well as clinical officers and associate medical officers (paraprofessionals with authority to diagnose and treat routine illness). Nursing classifications varied too much across countries to consistently distinguish these further; the category of nurse included all midwives. The final category included nursing aides, assistants and any other personnel (e.g. counsellors and social workers). Providers were considered to have a supportive environment if they reported at least one of the following: clear job description, knowledge of opportunities for promotion or availability of performance incentives.

Facility covariates included: ownership (private versus government) and measures of general service provision readiness (number of services provided; number of clinical staff per bed [small facilities without beds were assigned a value of one to permit comparison of staffing with larger facilities]; equipment availability; facility infrastructure; and facility management practices). For the last three measures we created indices composed of multiple items; details are available from the corresponding author. We calculated the natural log of the number of services offered by the facility and staff per bed for easier interpretation of the results. Finally, as this was a pooled analysis of all seven countries, we used an indicator variable for country as a proxy for national factors that may influence quality.

### Statistical analysis

To compare quality across countries, we calculated mean and interquartile range (IQR) for antenatal care and sick-child care quality. For each process quality score and explanatory covariate, we estimated the mean and standard deviation (SD), weighted based on client sampling weights. Bivariate analyses were then performed for quality on each covariate. Variables were included in the final model if they were statistically significant at the *P* < 0.10 level for at least one type of visit (antenatal care or sick-child visits) or were conceptually important. We estimated two-level random intercept regression models with visits nested within providers for each service. The large proportion of clinics with a single provider prevented construction of a three-level model (visit, provider and clinic). Estimates of between-provider difference thus include both facility differences and provider differences. Malawi served as the reference category as it was the poorest country in this study (Table 1). To test the impact of the Hawthorne effect on the results (a change in behaviour as a result of being observed^35^), we conducted sensitivity analysis without the first observation per provider within each service. More details are available from the corresponding author.

We calculated the percentage of variation in quality explained by the covariates as the difference in variance between the adjusted model and the null model divided by the null model variance. We quantified the explained variance for each group of covariates (country, facility, provider and visit) by progressively adding blocks of variables to the multilevel random intercept models. Regression analyses are unweighted due to adjustment for factors associated with respondent selection; models are clustered by facility.

All statistical analyses were carried out using Stata version 14.1 (StataCorp, College Station, United States of America).

## Results

Across the seven countries, 4613 of 4798 sampled facilities were successfully assessed (96.1%); 2902 of these facilities were primary-care facilities with at least one clinical observation in antenatal or sick-child care. A total of 2594 first antenatal care visits and 11 402 clinical consultations for children younger than 5 years were fully observed in primary-care facilities. These visits were to 3902 unique providers (1077 for antenatal care and 3144 for sick-child care; 319 providers were observed in both services).

Of the visits for antenatal care, 430 (16.7%) were by teenage women and over half (1268; 52.9%) by those presenting late for the first visit at that facility (Table 3). Of the observed sick-child visits, 4073 (35.1%) were for infants; the average child presented with close to three symptoms. Three-quarters of antenatal visits and almost half of sick-child visits were handled by nurses; only 74 (2.8%) of antenatal visits but 3565 (30.5%) of sick-child visits were dealt with by physicians or clinical/associate medical officers. Overall, 11 387 of 14 452 (78.8%) of patient visits were to public health-care facilities.

**Table 3:** Characteristics of 13 996 clinical observations at visits for antenatal and sick-child care in Kenya, Malawi, Namibia, Rwanda, Senegal, Uganda and the United Republic of Tanzania, 2006–2014

Characteristic	Antenatal care			Sick-child care	
	No. of observations, weighted	Value		No. of observations, weighted	Value
**Dependent variable**					
Clinical quality,^a^ mean (IQR) (%) score	2 638	62.2 (50.0 to 75.0)		11 814	54.5 (33.3 to 66.7)
**Visit variables**					
Afternoon visit, no. (%)	2 635	1 148 (43.6)		11 794	3 291 (27.9)
Education attainment secondary school or higher, no. (%)	2 636	497 (18.8)		11 764	2 587 (22.0)
First antenatal visit ≥ 24 weeks, no. (%)	2 398	1 268 (52.9)		N/A	N/A
Teenage antenatal patient, no. (%)	2 574	430 (16.7)		N/A	N/A
Age of sick child, no. (%)	N/A	N/A		11 605	11 605 (100)
< 12 months	N/A	N/A		–	4 073 (35.1)
12–60 months	N/A	N/A		–	7 532 (64.9)
Complaints per sick child, mean (SD) no.	N/A	N/A		11 783	2.77 (1.23)
**Provider variables**					
Cadre, no. (%)	2 593	2 593 (100)		11 689	11 689 (100)
Physician/clinical officer	–	74 (2.8)		–	3 565 (30.5)
Nurse/midwife	–	1 960 (75.6)		–	5 717 (48.9)
Nursing assistant/aide/other^b^	–	560 (21.6)		–	2 407 (20.6)
Completed pre-service education > 5 years before, no. (%)	2 568	1 511 (58.9)		11 637	6 696 (57.5)
Supportive environment,^c^ no. (%)	2 592	2 428 (93.7)		11 688	10 583 (90.5)
**Facility variables**					
Managing authority, no. (%)	2 638	2 638 (100)		11 814	11 814 (100)
Government	–	2 169 (82.2)		–	9 218 (78.0)
Private	–	469 (17.8)		–	2 595 (22.0)
Services in facility, mean (SD) no.	2 638	13.27 (3.10)		11 814	12.46 (3.40)
Staff per bed,^d^ mean (SD) no.	2 535	3.57 (4.13)		11 328	3.49 (4.88)
Infrastructure index,^e^ mean (SD)	2 638	0.56 (0.16)		11 814	0.56 (0.16)
Equipment index,^f^ mean (SD)	2 638	0.73 (0.19)		11 791	0.78 (0.26)
Management index,^g^ mean (SD)	2 638	0.65 (0.18)		11 814	0.63 (0.19)
**Country, no. (%)**	2 638	2 638 (100)		11 814	11 814 (100)
Kenya	–	344 (13.0)		–	1 516 (12.8)
Malawi	–	513 (19.5)		–	2 136 (18.1)
Namibia	–	363 (13.8)		–	1 430 (12.1)
Rwanda	–	350 (13.3)		–	1 583 (13.4)
Senegal	–	407 (15.4)		–	2 323 (19.7)
Uganda	–	146 (5.5)		–	704 (6.0)
United Republic of Tanzania	–	515 (19.5)		–	2 122 (18.0)

IQR: interquartile range; N/A: not applicable; SD: standard deviation.

^a^ Clinical quality scores are the percentage of recommended clinical actions done at the observed visit (eight items for antenatal care, nine for sick-child care). Score range is 0–100, with higher scores corresponding to greater adherence to the recommended clinical actions.

^b^ Other category includes counsellors and social workers.

^c^ Provider-supportive environment is a binary indicator that takes the value of one if any of three elements are present: clear job description, knowledge of opportunities for promotion, or availability of performance incentives.

^d^ Staff per bed adds one to the bed count in each facility to include facilities reporting zero beds in the analysis.

^e^ Infrastructure index is the proportion of 20 supply-side factors present in each facility, including the availability of a functional ambulance and uninterrupted essential drug supply over the past month.

^f^ Equipment index is the proportion of equipment essential for visits for antenatal care (seven items, e.g. functioning fetal stethoscope and weighing scale) and sick children (four items, e.g. functioning thermometer and stethoscope) observed in each facility.

^g^ Management index is the proportion of 10 indicators of facility management practices fulfilled in each facility, including regular quality assurance reviews and supervisory visits.

Notes: Data were pooled from service provision assessment surveys of health facilities in each country (survey year range: 2006–2014). Descriptive statistics were weighted using client sampling probabilities in the surveys. Total number of observations for antenatal care were 2594 unweighted, 2638 weighted; for sick-child care were 11 402 unweighted, 11 814 weighted.

**Fig. 1:**
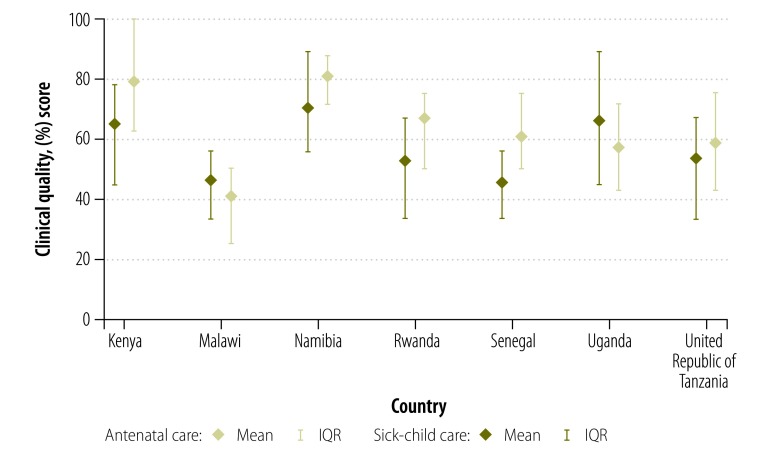
Range of clinical quality observed at visits for antenatal and sick-child care in Kenya, Malawi, Namibia, Rwanda, Senegal, Uganda and the United Republic of Tanzania, 2006–2014

**Fig. 2:**
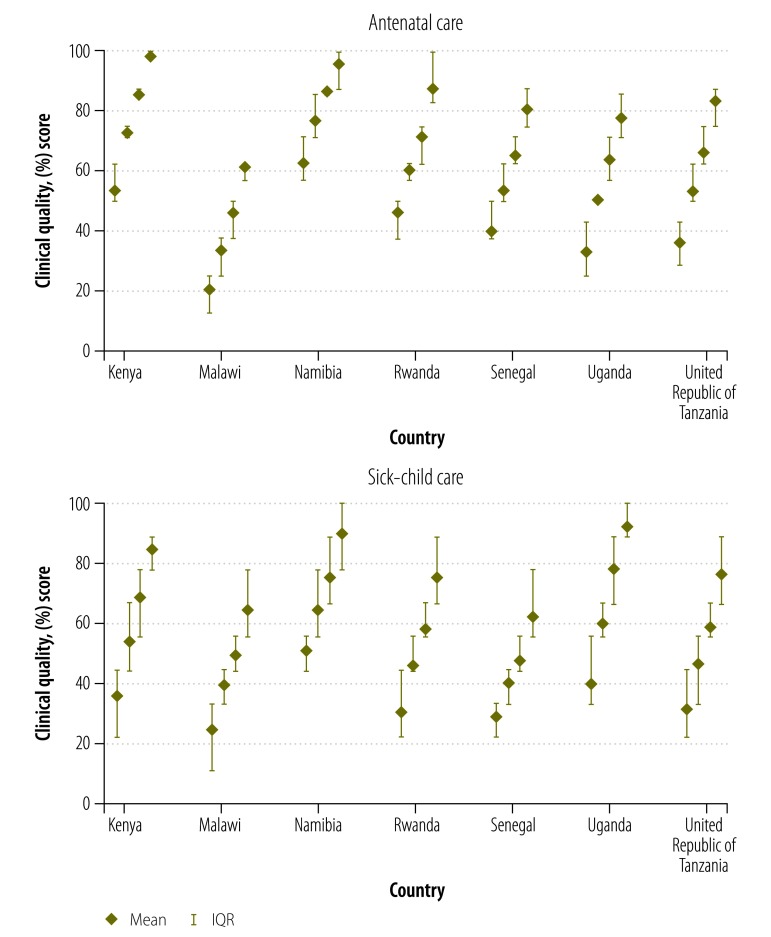
Variation in clinical quality observed at antenatal care visits and sick-child care visits, by quartile of provider quality in Kenya, Malawi, Namibia, Rwanda, Senegal, Uganda and the United Republic of Tanzania, 2006–2014

Overall quality of care was low, with a mean score of 62.2% (IQR: 50.0 to 75.0) for antenatal visits and 54.5% (IQR: 33.3 to 66.7) for sick-child care visits. Quality varied considerably across the countries surveyed, as shown in the comparison of quality scores by country in Fig. 1. The quality of care for pregnant women was typically higher than for sick children. Fig. 2 displays the variance in clinical quality when providers were grouped according to quartiles of average quality by country. Variability within quartiles was not consistently associated with average quality: both poor and good providers displayed considerable variability.

IQR: interquartile range.

Notes: Data were obtained from service provision assessment surveys of health facilities in each country (survey year range: 2006–2014). Data points show the mean and IQR clinical quality score of visits for each country. Quality scores are the percentage of recommended clinical actions done by health-care providers at the visit (eight items for antenatal care, nine for sick-child care); mean and IQR are calculated across all observed visits per country. Data for Kenya, for example, indicate that in an average visit, a provider completed 79.2% of items (6.3 of 8) for antenatal care and 64.9% of expected items (5.8 of 9) for sick-child care.

IQR: interquartile range.

Notes: Data were obtained from service provision assessment surveys of health facilities in each country (survey year range: 2006–2014). For antenatal care a total of 2594 visits were made to 1077 health-care providers; for sick-child care 11 402 visits were made to 3144 health-care providers (319 providers were observed in both services). Data points show the mean and IQR clinical quality score of visits for each country. Quality scores are the percentage of recommended clinical actions done by health-care providers at the visit (eight items for antenatal care, nine for sick-child care); mean and IQR are calculated across all observed visits per country.

Table 4 presents the results of the fully adjusted, multivariable, random intercept regression models. The analytical sample included 2173 antenatal visits (83.8%) and 10 646 sick-child visits (93.4%) with complete data on covariates. For antenatal care, higher-risk women received significantly worse care than other women (−1.9 percentage points out of 100 for teenage mothers and −1.6 percentage points for late first visits). Within providers, the only significant association was underperformance, relative to nurses, of the small number of clinicians providing antenatal care (−8.3 percentage points difference). Quality of care scores were higher at private facilities (4.5 percentage points better than public facilities), at facilities with more staff (2.0 percentage points increase for each doubling of staff per bed), and at facilities with better infrastructure and equipment (differences of 9.8 and 16.5 percentage points for a score of 1 versus 0 on these indices). Using Malawi as the reference, the six other countries had significantly higher antenatal care quality, with differences up to 33.4 percentage points for Kenya and 32.5 percentage points for Namibia, the two highest income countries in this study. The intra-class correlation in the unadjusted model was 81.4%, indicating relatively low variability in quality of care within providers (i.e. between visits).

**Table 4:** Results of multilevel regression models of clinical quality observed at visits for antenatal and sick-child care in Kenya, Malawi, Namibia, Rwanda, Senegal, Uganda and the United Republic of Tanzania, 2006–2014

Characteristic	Quality coefficient^a^ (95% CI)	
	Antenatal care (*n* = 2173)^b^	Sick-child care (*n* = 10 646)^b^
**Visit variables**		
Afternoon visit	−0.2 (−1.8 to 1.3)	−0.5 (−1.5 to 0.4)
Educational attainment above secondary school	0.6 (−0.9 to 2.1)	−0.9 (−1.7 to −0.03)
First antenatal visit ≥ 24 weeks	−1.6 (−2.7 to −0.5)	N/A
Teenage antenatal patient	−1.9 (−3.5 to −0.4)	N/A
Age of sick child		
< 12 months	N/A	2.0 (1.4 to 2.7)
12–60 months	N/A	Ref.
Complaints per sick child	N/A	2.6 (2.3 to 2.8)
**Provider variables**		
Cadre		
Physician/clinical officer	−8.3 (−13.4 to −3.1)	0.7 (−1.3 to 2.6)
Nurse/midwife	Ref.	Ref.
Nursing assistant/aide/other	−3.2 (−6.8 to 0.5)	−3.1 (−5.0 to −1.2)
Graduated > 5 years before	−1.2 (−3.6 to 1.3)	1.8 (0.6 to 3.1)
Supportive environment	−2.8 (−7.3 to 1.7)	0.3 (−2.1 to 2.7)
**Facility variables**		
Managing authority		
Government	Ref.	Ref.
Private	4.5 (1.2 to 7.8)	3.0 (1.4 to 4.7)
Services in facility (natural log of service count)	2.0 (−4.4 to 8.4)	−0.2 (−2.8 to 2.5)
Staff per bed (natural log of staff per bed)	2.9 (1.0 to 4.7)	0.2 (−0.8 to 1.1)
Infrastructure index	9.8 (0.7 to 18.8)	2.9 (−2.0 to 7.8)
Equipment index	16.5 (8.5 to 24.4)	2.6 (−0.1 to 5.3)
Management index	−1.9 (−9.3 to 5.6)	4.9 (1.2 to 8.7)
**Country**		
Kenya	33.4 (28.4 to 38.4)	15.7 (12.6 to 18.8)
Malawi	Ref.	Ref.
Namibia	32.5 (27.8 to 37.1)	26.0 (23.4 to 28.7)
Rwanda	23.2 (18.6 to 27.9)	6.5 (3.9 to 9.1)
Senegal	18.8 (13.5 to 24.0)	1.2 (−1.2 to 3.6)
Uganda	14.4 (9.2 to 19.6)	22.1 (18.8 to 25.3)
United Republic of Tanzania	18.5 (13.4 to 23.7)	8.9 (6.4 to 11.4)
**Intercept**	22.4 (3.1 to 41.7)	30.0 (22.5 to 37.5)
**Total variance**	330.4	397.6
**Provider variance**	232.5 (206.9 to 261.3)	204.5 (191.2 to 218.7)
**Residual variance**	98.0 (84.3 to 113.8)	193.1 (185.2 to 201.3)

CI: confidence interval; N/A: not applicable; Ref.: reference category.

^a^ Quality coefficient is the expected difference in visit quality (scale 0 to 100) given a 1 unit difference in the exposure, holding all other covariates constant.

^b^ *n* is the number of observations with complete data on covariates.

Notes: Data were pooled from service provision assessment surveys of health facilities in each country (survey year range: 2006–2014). All standard errors are clustered by facility. Information on indices (e.g. provider support, infrastructure) is in the notes to Table 3. Intraclass correlation between visits for providers in the unadjusted model was 81.4% for antenatal care and 59.0% for sick-child care.

Results for quality of sick-child care differed in several ways (Table 4). Higher-risk children, i.e. infants and those with more symptoms, received better care (differences of 2.0 and 2.6 percentage points, respectively) than other children. Physicians provided similar quality of care relative to nurses, with assistants and aides significantly worse than nurses (−3.1 percentage points). More-experienced providers provided significantly higher quality care. Of facility characteristics, only private facilities and better management practices were significantly associated with higher quality of care. All other countries except Senegal provided higher quality care on average than Malawi, notably Namibia and Uganda (> 20 percentage points higher). The intraclass correlation in the unadjusted model was 59.0%, evidence of moderate between-visit variability in providers’ quality of care.

Overall, the full models explained 37% of the total variance in antenatal care and 20% of the variance in sick-child care. Over 80% of explained variance in each service was due to the country variable. Only facility characteristics for antenatal care (19% of explained variance) and visit characteristics for sick-child care (10% of explained variance) contributed meaningfully to the model’s explanatory power. Findings for both services were largely unchanged in sensitivity analysis excluding first observations. Variance estimates and sensitivity analysis are available from the corresponding author.

**Fig. 3:**
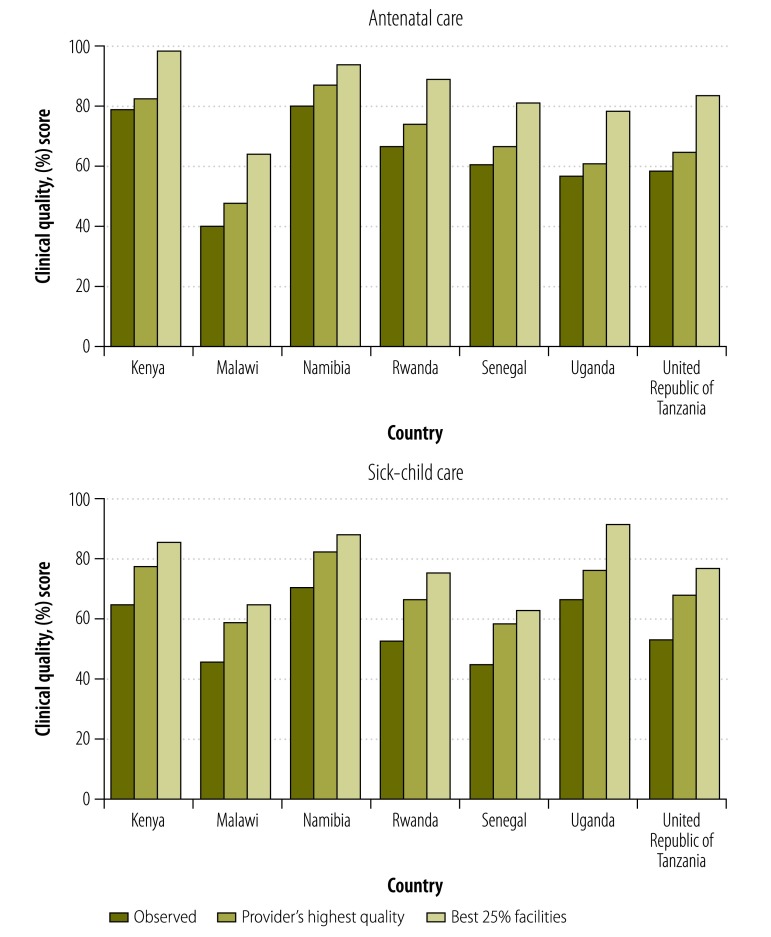
How clinical quality would change if all providers performed at their highest observed level and at the level of the highest quartile of facilities in Kenya, Malawi, Namibia, Rwanda, Senegal, Uganda and the United Republic of Tanzania, 2006–2014

Notes: Data were obtained from service provision assessment surveys of health facilities in each country (survey year range: 2006–2014). The three bars represent: (i) the level of quality of care measured in this study; (ii) the predicted quality if all consultations were at the provider’s highest quality visit; and (iii) the predicted quality if all consultations were done to the same standard as the top 25% of primary-care facilities in the country.

Fig. 3 depicts the scope for improvement in quality in each country. Enabling providers to provide quality of care at their own peak performance would result in gains of over 5% in antenatal care quality and 10% in sick-child care quality. Bringing all visits up to the standard of the top quartile of facilities would result in linear increases of over 20% in quality of care in both services across all countries.

## Discussion

In this analysis of nearly 14 000 clinical consultations in seven countries, we found relatively weak quality of care for pregnant women and sick children: providers performed half to two thirds of a minimal set of recommended clinical actions. Providers for antenatal care were primarily nurses, whereas sick children were seen by both nurses and clinical officers. Nursing assistants conducted one in five visits for both services. Other studies in similar settings, often done in the context of quality improvement, have found that the care of sick children was weak.^36^^,^^37^

Performance differed substantially across countries, not only due to differences in national wealth or health-worker supply. For example, Kenya’s average antenatal care quality was comparable to Namibia’s despite having half the per capita number of physicians and one-quarter the national income. Uganda, with one-tenth the national income and one-third the physicians of Namibia, performed nearly as well in sick-child care. Quality was not consistent across services; countries with strong performance in antenatal care did not always do well in sick-child care. Factors such as national leadership and governance, health-care budget allocation, financing schemes, the role of donors, geography and the quality of basic and medical education are likely to be important in explaining the differences.^38^ While there are no comparable cross-national, quality data from lower-income countries, data from the Organisation for Economic Co-operation and Development also show heterogeneous performance that is not linked to national wealth or health-system resources in high-income countries.^39^ This is an important area for further study, as country indicators accounted for the majority of the explained quality variation in our analysis.

We analysed a large range of factors previously found to influence quality of care. Antenatal care quality was strongly associated with facility factors: staffing, infrastructure and equipment. In contrast, sick-child care was related not to facility equipment but to better facility management. Private facilities outperformed public facilities for both services, even after controlling for a range of facility inputs and management practices. We were unable to assess for-profit and not-for-profit facilities separately as not all countries collected these data; this would be an important area for future work.

Higher qualifications of health-care providers did not guarantee better care. Clinical officers and physicians performed substantially worse than nurses in antenatal care and did no better than nurses in sick-child care. Previous research has shown mixed results when comparing nurses with other health-care professionals.^40^^,^^41^ Nursing assistants and aides were not as competent in sick-child care as physicians and nurses. More experienced health-care providers performed better in sick-child care. Although we included a variable for supportive job environment, including opportunities for promotion, we did not have information on intrinsic motivation, quality of supervision or remuneration, any of which may have influenced performance.^34^

While the majority of the variation in performance (81.4% for antenatal care and 59.0% for sick-child care) stemmed from differences in quality of care across providers (including their country and clinic factors), individual providers also gave different care to different patients. Patient and visit factors were influential in explaining the quality of sick-child care in particular. Care quality was higher for younger and sicker children. Antenatal care was weaker for teenage patients and those presenting after 24 weeks of pregnancy (potentially due to prior antenatal care). While a patient’s specific presentation and case severity can alter providers’ clinical actions in a consultation, the items included in our quality indexes represented basic medical procedures that should have been done for all patients.^42^

The best performance by providers and clinics in each country suggest considerable scope for national improvement in quality. All countries in this study could make large gains in quality if providers performed at their best and if all facilities performed at the level of the top quarter of clinics. The visit-to-visit variation within individual providers may be decreased by better adherence to guidelines and intensive supervision to promote more consistent performance of essential functions.

This analysis has several limitations. Although direct observation is the gold standard of clinical quality measurement, it is subject to the Hawthorne effect and observer error. We did not find evidence that the Hawthorne effect materially influenced the intra-provider variation within the relatively small number of observations per provider.^35^ However, we cannot rule out mistakes in the observers’ recording of clinical care contributing to between-visit variance; some researchers consider between-visit variation a nuisance parameter reflecting statistical noise.^43^ Our quality indices were defined based on items asked in all service provision assessment surveys and matched with evidence-based guidelines. While other items could be added to provide a more complete assessment of quality, these indices represent a minimal level of quality. We were not able to link the process of clinical care to patient outcomes. Measurement of provider and facility characteristics differed across the service provision assessment surveys. For example, we were not able to investigate differences within classifications of nurses due to lack of disaggregated data in some countries. The data did not contain contextual factors that may contribute to variation in the processes of care, such as local epidemiology, and community factors that may influence clinic performance, such as accountability charters or strong local district management. To limit the role of such factors in this analysis, we constructed quality metrics limited to only the most essential clinical functions. In future, it would be valuable to assess more aspects of compliance with WHO guidelines^32^^,^^33^ on clinical care for mothers and infants. Finally, the service provision assessment contains observations for only a few services. Whether quality differs for other primary-care services should be explored.

Analysis of variations in quality of care processes can lay the groundwork for quality improvement.^44^ Equipment, staffing and management factors affected quality of care and these provide concrete areas for improvement. However, the substantial variation in quality of care across the study countries after accounting for these measured factors should prompt examination of national standards for professional education of health-care providers and health-system policies to support quality care. The finding that quality also varied across clinics in the same country and even among consultations done by the same provider suggests that identifying and replicating local best practices will be valuable. Efforts are under way to design better models of antenatal care and to test innovations in primary care.^45^^–^^47^

The first step to closing the quality gap is to measure it. Governments of lower-income countries that want to enhance their health outcomes and provide better services to citizens can use these data as a baseline for improvement. Global partners should support the means to fund comparative analyses, develop efficient measures, assist in improving of routine information systems, and train local health system researchers. Reaching the SDGs will require a shared commitment to this new agenda.
